# From the bench to exploration medicine: NASA life sciences translational research for human exploration and habitation missions

**DOI:** 10.1038/s41526-016-0002-8

**Published:** 2017-01-12

**Authors:** Joshua S. Alwood, April E. Ronca, Richard C. Mains, Mark J. Shelhamer, Jeffrey D. Smith, Thomas J. Goodwin

**Affiliations:** 1 Space BioSciences Division, NASA Ames Research Center, Moffett Field, CA USA; 2 Wake Forest School of Medicine, Winston-Salem, NC USA; 3 Mains Associates, Berkeley, CA USA; 4 Human Research Program, NASA Johnson Space Center, Houston, TX USA; 5 Biomedical Research and Environmental Sciences Division, NASA Johnson Space Center, Houston, TX USA

## Abstract

NASA’s Space Biology and Human Research Program entities have recently spearheaded communications both internally and externally to coordinate the agency’s translational research efforts. In this paper, we strongly advocate for translational research at NASA, provide recent examples of NASA sponsored early-stage translational research, and discuss options for a path forward. Our overall objective is to help in stimulating a collaborative research across multiple disciplines and entities that, working together, will more effectively and more rapidly achieve NASA’s goals for human spaceflight.

## Introduction

With the International Space Station (ISS) available for research and habitation for the next decade, there is now a precious opportunity to significantly advance the contributions of space life sciences and space medicine toward enabling the Exploration Class missions (that is, human missions beyond low-Earth orbit). Such missions are currently burdened with significant anticipated crew health and safety challenges.^1^ Coordinated research and development are crucial for producing effective countermeasures against the deleterious influences of spaceflight on the human body. To this end, we emphatically support the development of translational research efforts at NASA that will facilitate bi-directional information flow between basic and applied life sciences and medical operations with the goal to accelerate countermeasure development.

As identified by National Academy committees and embedded in the NASA Strategic Plan, a sustainable human spaceflight program requires robust research at both *basic* and *applied* levels to make fundamental biological discoveries and to address the known and emerging risks to human health resulting from the spaceflight environment, respectively.^2–5^ The Life Sciences portfolio at NASA is composed of the Space Biology program (a basic science program), the Human Research Program (HRP, an applied program), and Medical Operations (Office of Chief Health and Medical Officer). Informal translational research within these programs has taken place at NASA for decades, yet there is a need for a more coordinated effort that enables dynamic translation that can take rapid advantage of new findings. In particular, the National Academy committees recommended that NASA follows key elements of the National Institutes of Health’s (NIH) model of translational science.^6,7^ It was specifically recommended that the *coordination* and *maturation* of projects between each level of research be enhanced to increase knowledge transfer and identification of potential solutions for astronaut health challenges.^2–4^ Additionally, the translation of knowledge to Earth-based medical care has recently begun with the designation of the ISS as a National Laboratory (currently through the Center for the Advancement of Science in Space).

**Fig. 1:**
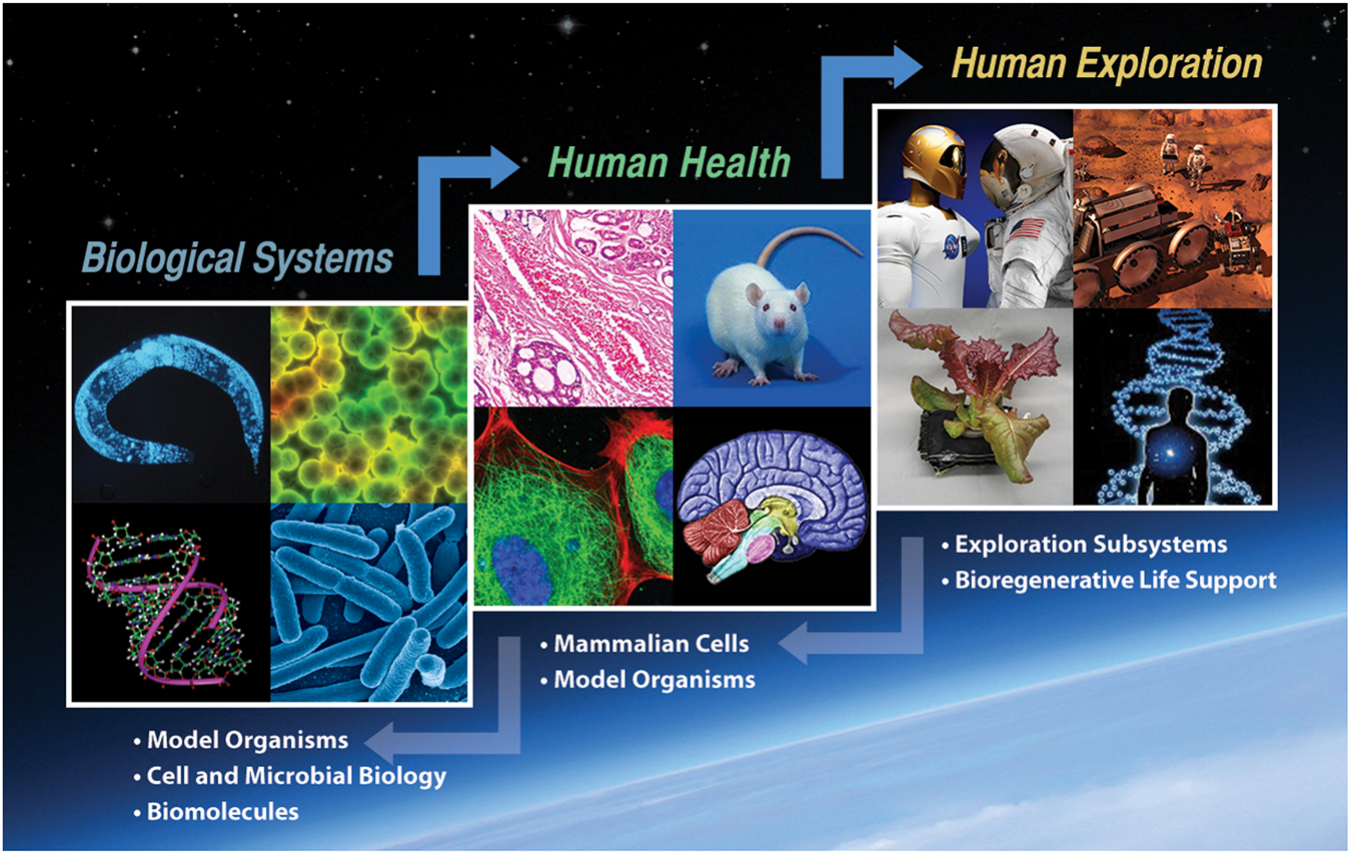
The NASA Life Sciences Translational Path. It moves from basic research to human exploration applications with bi-directionality options. As knowledge is applied along this path (*top arrows*) questions may arise that can best be addressed by more basic research (*bottom arrows*) in order to support further progress toward successful human exploration

In this paper, we provide context for the current drivers (and needs) for translational research at NASA, briefly summarize the experience at NIH, and lay out a two-phased approach for translational research. As illustrated in Fig. 1, the first phase is *basic-to-applied* and the second phase is *applied-to-operational*. We define the levels of the research continuum as follows, consistent with the Decadal Survey’s Interim Report:^5^ *basic research* aims to generate new biological knowledge using biological systems or model organisms, *applied research* aims to develop countermeasures for risks to astronaut health (similar to pre-clinical research), and *medical operations* puts countermeasures into practice for astronauts (like operations in the clinic).

Finally, going into greater depth on the *basic-to-applied* window of translation, we provide examples and recommendations of early-stage research where Space Biology and HRP aim to build synergy between their existing programs. NASA’s Space Biology and HRP entities have recently spearheaded communications both internally and externally to coordinate the Agency’s translational research efforts between basic and applied levels. In this paper, we strongly advocate for translational research at NASA, provide recent examples of NASA-sponsored early-stage translational research, and discuss options for a path forward. Our overall objective is to help in stimulating the collaborative research across multiple disciplines and entities that, working together, will more effectively and more rapidly achieve NASA’s goals for human spaceflight.

## NASA’s involvement in translational research

### Definitions

For the purposes of this paper we will focus mainly on the translation of space life sciences research knowledge to the human challenges associated with long-duration spaceflights. Our narrow translational research focus is therefore defined as the application of basic science discoveries to the development of new preventive and therapeutic approaches supporting optimized human adaptation to space missions with extended durations and distances from Earth.

### NASA’s human spaceflight challenges

The biomedical concerns and risks of human Exploration Class missions, characterized by extended duration space exposures and travel far beyond Earth, are not yet fully understood.^8^ Prior to an exploration mission, better definition of these risks, and the development and implementation of countermeasures must be achieved to avoid physiological and behavioral changes that could degrade the health, safety and productivity of crew members during extended ISS habitation and missions beyond Low Earth Orbit (LEO). Additionally, during exploration missions, tools are needed to monitor and manage individual and crew resiliency in the face of unplanned challenges.^9^

The ISS presently accommodates continuous human habitation, enabling basic and applied research and testing of new ways to thrive in space. ISS offers a unique platform that is facilitating the identification of requirements for conducting future human missions that could last for years, and the challenges associated with orbiting and even occupying other planets. Exploration Class missions beyond ISS will require detailed knowledge of what adaptations can be tolerated and those which must be countered for the long-term. These missions will also require a degree of operational resiliency and autonomy never before attempted. Human space exploration is now at a crossroads, and leaving LEO will place increasing demands on space life sciences research to support such endeavors.

Key spaceflight-related environmental factors that chronically impact human health and performance during such missions are: microgravity, confinement, and exposure to space radiation.^1^ We chose just four examples, profiled below, to demonstrate the value of translational research in addressing four specific risks identified by NASA for astronaut health and safety: immune response, microbe-host interactions, oxidative stress and damage, and visual impairment (VI) syndrome. There are many others on NASA’s list,^10^ of course, including some in broad areas such as nutrition and sleep, and surely some not yet identified. For NASA, translational research has historically been^11^ and is increasingly viewed as essential to better define and minimize these health risks.

### Translational research: pioneered by NIH

NIH has pioneered the translational research challenge since about 2003.^7^ Several NIH institutes have been established under the National Center for Advancing Translational Sciences^6^ to develop a “Translational Pipeline” linking basic research to pre-clinical and clinical investigations^7,12^ that will lead to eventual utilization in the provision of health care. Both the NIH and NASA research portfolios cover a broad range of spatial scale and complexity, including microbiology, cell culture, model organisms, and an array of human biomedical studies (Fig. 1). NASA can learn much from NIH’s endeavors, and the implementation of cooperative agreements between these agencies could provide effective bridges for knowledge sharing.

### Recent NASA drivers toward translational research

In 2009, after consultation with NASA, Congress determined that an objective reassessment was needed of the basic and applied research capabilities and resources available to support future human space exploration. Congress mandated that the National Research Council conduct and publish a “Decadal Survey” ultimately titled *Recapturing a Future for Space Exploration: Life and Physical Sciences Research for a New Era*.^3^ The Decadal Survey reviewed the research, development, and applications relevant to basic science and human space exploration. The Space Life and Physical Sciences research communities provided major input to the Decadal Survey and helped to shape its many broad recommendations. A key recommendation was to increase the integration of efforts toward conduct of cross-disciplinary, focused translational research and development with applications to human space risks and related areas supporting human space exploration. Another main recommendation was for NASA to constitute an integrated organization within NASA to manage these efforts. In 2011, the Space Life and Physical Science Research & Applications Division was established within NASA’s Human Exploration and Operations Mission Directorate.

**Fig. 2:**
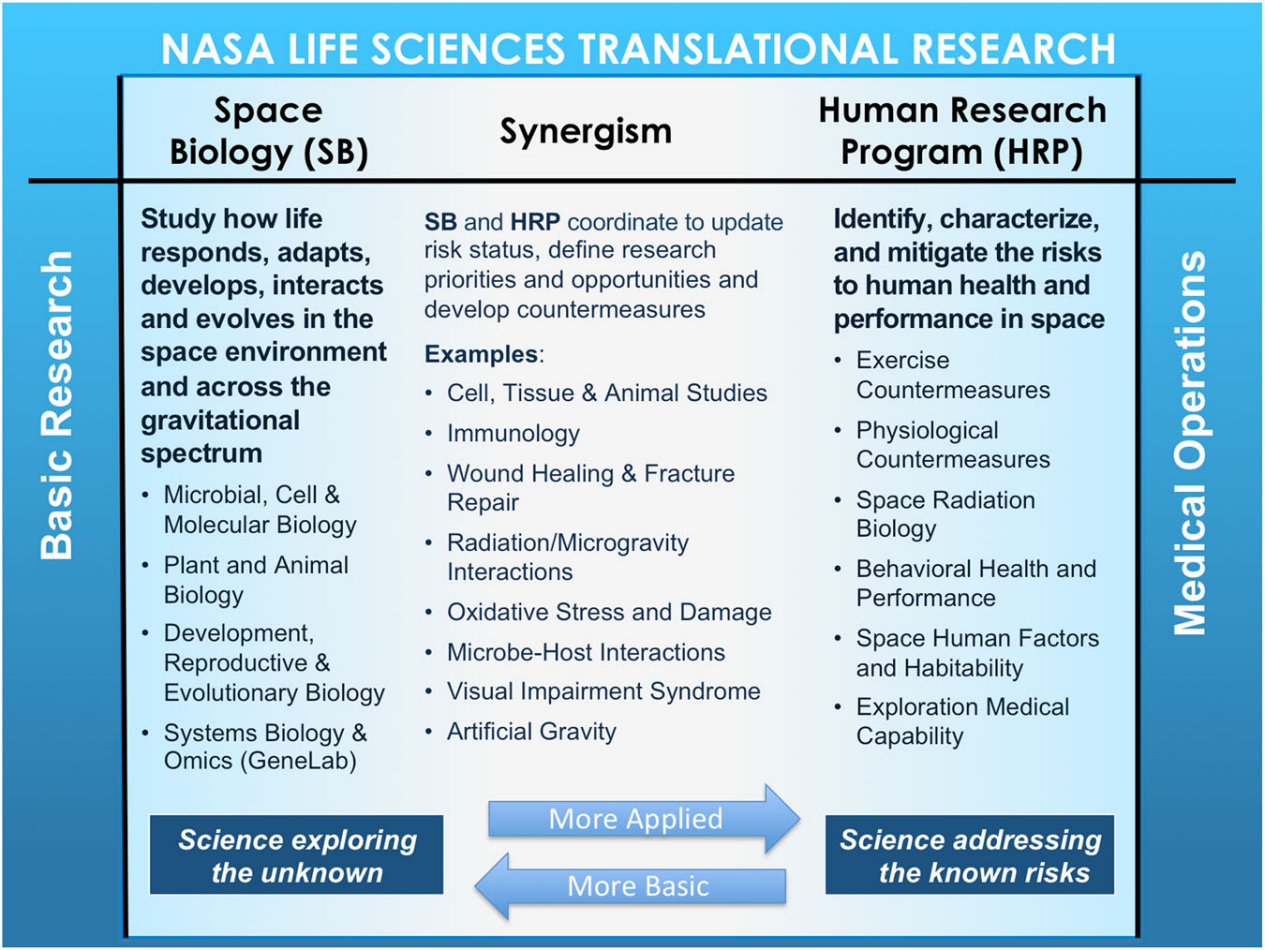
Space Biology and Human Research Bi-directional Synergism. Basic Space Biology research is done mainly with lower-level model organisms, cells, and tissues. The HRP conducts biomedical science mainly with humans. They synergistically and bi-directionally collaborate in maximizing opportunities for translating a subset of that knowledge to optimize the health and safety of the crew via applications supporting medical operations

Key life science elements within the SLPSRA Division include the HRP and the Space Biology program (Fig. 2). HRP created and maintains a dynamic Human Research Roadmap that defines known risks, mitigation capabilities, current knowledge gaps, and concepts for future work regarding astronaut health, performance, and safety.^10^ In contrast, Space Biology focuses on defining the changes in basic biological mechanisms as a result of spaceflight and has developed a set of Guiding Questions that define its research program.^13^ Despite the programmatic differences, there are overlapping research areas within these two programs at the *basic-to-applied* phase of translation, which are itemized in Fig. 2 and described below in the Examples section.

The Decadal Survey panels made recommendations for research priorities throughout the lifespan of the ISS. In Chapter 6 of the Decadal Survey, the *Animal and Human Biology* panel (3) made 16 key recommendations spanning multiple systems in the body of vertebrates for conduct of basic and applied research (Table 1). The panel indicated that many of these should be studied using a translational framework. Further, the panel unanimously stated that animal research be firmly integrated in the space life sciences portfolio—both within space-based and ground-based programs—to drive the translation of knowledge. Additionally, in Chapter 7 of the Decadal Survey, the *Integrative and Translational Research for the Human Systems* panel (3) made 11 recommendations related to crosscutting issues (CC) for humans in the space environment (Table 2). One goal of this paper is to bring stakeholders—U.S. taxpayers, Congress, the National Academies, and relevant science communities—up to date regarding NASA’s progress in building collaborative research communities in the translational area to pursue these important recommendations.

**Table 1:** Animal and human biology (AH) research recommendations for humans in the space environment identified in chapter 6 of the decadal survey (3)

Identifier	Recommendation
AH1	The efficacy of bisphosphonates should be tested in an adequate population of astronauts on the ISS during a 6-month mission.
AH2	The preservation/reversibility of bone structure/strength should be evaluated when assessing countermeasures.
AH3	Bone loss studies of genetically altered mice exposed to weightlessness are strongly recommended.
AH4	New osteoporosis drugs under clinical development should be tested in animal models of weightlessness.
AH5	Conduct studies to identify underlying mechanisms regulating net skeletal muscle protein balance and protein turnover during states of unloading and recovery.
AH6	Conduct studies to develop and test new prototype exercise devices and to optimize physical activity paradigms/prescriptions targeting multisystem countermeasures.
AH7	Determine the daily levels and pattern of recruitment of flexor and extensor muscles of the neck, trunk, arms, and legs at 1 g and after being in a novel gravitational environment for up to 6 months.
AH8	Determine the basic mechanisms, adaptations, and clinical significance of changes in regional vascular/interstitial pressures (starling forces) during long-duration space missions.
AH9	Investigate the effects of prolonged periods of microgravity and partial gravity (3/8 or 1/6 g) on the determinants of task-specific, enabling levels of work capacity.
AH10	Determine the integrative mechanisms of orthostatic intolerance after restoration of gravitational gradients (both 1 and 3/8 g).
AH11	Collaborative studies among flight medicine and cardiovascular epidemiologists are recommended to determine the best screening strategies to avoid flying astronauts with subclinical coronary heart disease that could become manifest during a long-duration exploration-class mission (3 years).
AH12	Determine the amount and site of the deposition of aerosols of different sizes in the lungs of humans and animals in microgravity.
AH13	Multiple parameters of T cell activation in cells should be obtained from astronauts before and after re-entry to establish which parameters are altered during flight.
AH14	Both to address the mechanism(s) of the changes in the immune system and to develop measures to limit the changes, data from multiple organ/system-based studies need to be integrated.
AH15	Perform mouse studies of immunization and challenge on the ISS, using immune samples acquired both prior to and immediately upon re-entry, to establish the biological relevance of the changes observed in the immune system. Parameters examined need to be aligned with those in humans influenced by flight.
AH16	Studies should be conducted on transmission across generations of structural and functional changes induced by exposure to space during development. Ground-based studies should be conducted to develop specialized habitats to support reproducing and developing rodents in space.

Reprinted with permission from Recapturing a Future for Space Exploration: Life and Physical Sciences Research for a New Era (2011) by the National Academy of Sciences, Courtesy of the National Academies Press, Washington D.C.

**Table 2:** Crosscutting Issues (CC) for Humans in the Space Environment identified in Chapter 7 of the Decadal Survey (3)

Identifier	Recommendation
CC1	To ensure the safety of future commercial orbital and exploration crews, quantify post-landing vertigo and orthostatic intolerance in a sufficiently large sample of returning ISS crews, as part of the immediate post-flight medical exam.
CC2	Determine whether AG is needed as a multisystem countermeasure and whether continuous large radius AG is needed or intermittent exercise within lower-body negative pressure or short-radius AG is sufficient. Human studies in ground laboratories are essential to establish dose-response relationships, and what gravity level, gradient, rotations per minute, duration, and frequency are adequate.
CC3	Conduct studies on humans to determine whether there is an effect of gravity on micronucleation and/or intrapulmonary shunting or whether the unexpectedly low prevalence of decompression sickness on the space shuttle/ISS is due to underreporting. Conduct studies to determine operationally acceptable low suit pressure and hypobaric hypoxia limits.
CC4	Determine optimal dietary strategies for crews and food preservation strategies that will maintain bioavailability for 12 or more months.
CC5	Initiate a robust food science program focused on preserving nutrient stability for 3 or more years.
CC6	Include food and energy intake as an outcome variable in dietary intervention trials in humans.
CC7	Conduct longitudinal studies of astronauts for cataract incidence, quality, and pathology related to radiation exposures to understand both cataract risk and radiation-induced late tissue toxicities in humans.
CC8	Expand the use of animal studies to assess space radiation risks to humans from cancer, cataracts, cardiovascular disease, neurologic dysfunction, degenerative diseases, and acute toxicities such as fever, nausea, bone marrow suppression, and others.
CC9	Continue ground-based cellular studies to develop end points and markers for acute and late radiation toxicities, using radiation facilities that are able to mimic space radiation exposures.
CC10	Expand understanding of gender differences in adaptation to the spaceflight environment through flight- and ground-based research, particularly potential differences in bone, muscle, and cardiovascular function and longterm radiation risks.
CC11	Investigate the biophysical principles of thermal balance to determine whether microgravity reduces the threshold for thermal intolerance.

Reprinted with permission from Recapturing a Future for Space Exploration: Life and Physical Sciences Research for a New Era (2011) by the National Academy of Sciences, Courtesy of the National Academies Press, Washington DC

## NASA’s current translational research approach

Several recent NASA-sponsored activities demonstrate an increased Agency commitment to two phases of translation (Fig. 1). Within the *basic-to-applied* phase, a collaborative workshop was held by NASA JSC and ARC in 2004 that addressed “Animal Research in Support of Human Space Exploration” before the translational research terminology was commonly used.^14^ Further, several recent events have been held to systematically advance NASA’s translational research discussions within the basic and applied space life sciences communities, including: an Artificial Gravity Workshop (responsive to the recommendations AH9-10 in Table 1 and CC2 in Table 2);^15^ symposia at the American Society for Gravitational Space Research (ASGSR) annual meetings in 2014^16^and 2015;^17^ and the annual HRP Investigators Workshops in 2015 and 2016. Finally, an internal analysis of the Space Biology and HRP research plans has pointed to areas, described in the examples section below and in Fig. 2, that are ripe for synergism.

Within the *applied-to-operational* phase, HRP established a Translational Research Institute, in October 2016, with a key goal to recruit funded, terrestrial biomedical research entities in furthering NASA translational research goals,^18–20^ and to help mature countermeasures and technologies for use in medical operations. Taken together, these efforts demonstrate new activities to facilitate translation from basic science to operations.

But how can translational research most effectively be pursued using existing resources? Team science is one solution^21^ and has been used successfully by NASA in the past. Additionally, enhanced institutional coordination within NASA and between federal funding agencies, as well as relevant non-governmental organizations, is needed to spur translation. A systematic approach to identify collaborative translational opportunities, for example by text analytics,^22^ would have benefits for investigators as well as NASA program managers. Text analytics is one method to systematically and automatically examine published papers for linkages based on common concepts (manifest in authorship, keywords, references, methods). This approach would allow the visualization of domains where research has been explored jointly within animals and humans or where the gaps in knowledge reside.

The Decadal Survey (3) provides guidance on how to structure a fully-integrated translational program by partitioning the life sciences research portfolio into two dimensions. *Horizontal integration* of projects is the first dimension and requires multidisciplinary approaches to solve complex problems, often within a given organism type. The second dimension, *vertical translation*, requires meaningful interactions among basic, applied, and operational scientists to translate fundamental discoveries to human treatments, such as using cell biology and model organism research to address human problems, for example in immune system response challenges.

The NIH experience has shown that the horizontal integration of research knowledge is essential for developing a comprehensive understanding of physiology and to effectively solve health problems.^23^ NASA has a successful record in this domain that should continue, for example, with its BioSpecimen Sharing Programs. Researchers have recently shared tissues from specific animals on a late Shuttle mission (STS-131),^24–31^ and the most recent Russian Bion biosatellite mission;^32–35^ sharing was in the form of preserved tissues from NASA’s Life Sciences Data Archive.^36^ Further, new Omic-science studies like NASA’s emerging GeneLab open-science data and biosample repository^37^ and the recent astronaut Twins Study^38^ enable big-data collection, analytics, and horizontal integration. Finally, restoring the gravity vector artificially via chronic whole-body centrifugation may solve issues in multiple tissues, simultaneously.

In contrast, vertical translation requires that investigators along the research continuum from basic to operational (Fig. 1) interact regularly to share ideas. Examples of vertical translation at NASA are in the recent International Life Sciences Research Announcement awards (NASA, European, Japanese, and Canadian space program research solicitations),^39^ GeneLab,^37^ and the recent call for proposals in the area of artificial gravity (AG).^40^ All of these planned research projects span multiple levels of the translational pipeline. Additionally, vertically-integrated team science occurs through NASA Specialized Centers of Research (NSCOR)^41–44^ and the National Space Biomedical Research Institute’s centers for radiation research,^45–47^ which aim to identify and translate countermeasure candidates for radiation-induced insults (Table 2: CC7–CC9). There is surely untapped value to be derived from additional vertical translation within NASA programs. Some existing overlap and synergism between HRP and Space Biology goals and approaches amenable to both horizontal and vertical translation are shown in Fig. 2.

Informally, vertical translation has occurred at NASA for many years^11^ with a conceptual maturation path shown in Fig. 1. For example, molecular mechanisms underlying the bone loss resulting from musculoskeletal disuse have been demonstrated in ground-based models,^48–50^ responding to recommendations AH2-4 (Table 1). Further, two precursors to osteoporosis drugs have been tested in space-flown or ground-based mice and shown to be effective at preventing weightlessness-induced bone loss,^49–51^ responsive to recommendations AH2, AH4 (Table 1). Thus, these drugs give HRP and Medical Operations additional potential countermeasures besides exercise^52^ and Alendronate^53^ to combat bone loss during spaceflight. Additional specific examples of vertical translation are described in the examples below.

## Examples of NASA life sciences basic-to-applied translational research

Progress in four specific areas is profiled below, each of which is a NASA “Health and Human Performance Risk for Space Exploration”.^8^ All demonstrate linkages between basic and applied research and involve on-going collaborations between Space Biology and the HRP. These examples illustrate ongoing opportunities for synergy between basic and applied researchers and these NASA programs to enhance and accelerate health-risk reduction.

### Immune response

The immune system is constantly adapting and is particularly responsive to unique environments such as those in spaceflight, especially for exposures of the long durations required by exploration missions beyond LEO. Immune system dysregulation (decreased responsiveness) has been seen during and after spaceflight and ground-space analog tests by studying humans,^54^ animals,^55^ and relevant cell cultures^56^ and are a priority area for study (AH13-15, Table 1). The specific causes are not yet clear, but are likely linked to one or more of the following factors: physiological stress, circadian rhythm disruption, microgravity exposure, isolation, altered nutrition, or radiation exposure.^57^ Further, the spaceflight environment compounds crew health risks as some microorganisms become more virulent (see below) and resistant to antibiotic drugs.

Even though the reactivation of latent viruses has been well documented in crewmembers,^58^ it is still unclear if the compromised immune response can lead to increased susceptibility to disease. There is not a sufficiently accurate ground-based analog to study immune suppression from spaceflight. However, extreme occupational environments such as Antarctica winter-over and the Aquarius undersea station enable aspects of immune dysregulation to be studied under similar stressors. Additionally, many valuable analog studies with animals and cells have been conducted, including unloading of rodents,^59^ and cell cultures and bioreactors.^56,60^ These studies have investigated immune response mechanisms and can allow the use of controlled diets, increased radiation levels and other factors that are not possible in human research.

### Microbe-host interactions

Preventive measures limit the presence of many medically significant microorganisms during spaceflight missions, but microbial infection of crewmembers cannot be completely prevented. Spaceflight experiments have demonstrated unique and heterogeneous microbial responses in spaceflight ecosystems and cultures,^61,62^ although the mechanisms behind those responses and their operational relevance remain unclear. In 2007, the operational importance of these microbial responses increased, as the results of Space Biology experiments aboard STS-115 and STS-123 demonstrated that an enteric pathogen (*Salmonella typhimurium*) increased in virulence in a mouse model of infection,^63,64^ responding to recommendation AH15 (Table 1). These studies can improve our understanding of the potential consequences to astronauts of elevated microbial virulence during long-duration missions.

Evidence for increased microbial virulence has recently been collected and reported from both spaceflight-analog systems and actual spaceflight.^61,65,66^ Although the conduct of virulence studies during spaceflight is challenging and often impractical in humans, data are being collected as part of the ISS Microbial Observatory,^62^ recent astronaut and rodent microbiome studies,^67–69^ and the edible plant studies (e.g., Veggie).^70,71^ When available, these results can improve our understanding of the astronaut-microbe interaction and of the potential health risks to the astronaut.

### Oxidative stress

The novel environmental conditions of spaceflight, and their combination, may affect both the generation and safe processing of reactive oxygen or nitrogen species.^72,73^ Evidence for oxidative-related issues in astronauts (or their analogs) can be found in the HRP Evidence Reports covering inadequate nutrition,^74^ extravehicular activity,^75^ and exposure to ionizing radiation.^47,76–79^ NASA has successfully used horizontally-integrated team science to understand and mitigate components of radiation-induced oxidative stress^42–44^ in mice.

### Visual impairment/intracranial pressure (VI/IP)

During and after long-duration spaceflights, some astronauts have reported noticeable, persistent VIs accompanied by ophthalmic changes including globe flattening, choroidal folds, optic-disc edema, and optic-nerve kinking.^80^ To date, clinically significant changes have been observed in male, but not female, astronauts,^81^ identifying a not yet understood sex difference, CC10 (Table 2). Increased intracranial pressure and optic-disc swelling (papilledema) may underlie these potentially irreversible changes.^81^ Current research is analyzing the effects of lowering cranial hypertension by using lower body negative pressure in astronauts during flight and bedrest,^82^ supporting the CC2 goal (Table 2).

Clinical signs and symptoms observed in astronauts have informed and focused important mechanistic studies exemplifying reciprocal translation at NASA (i.e., applied to basic). For example, retinas of female mice flown on the shuttle (STS-133, STS-135), acquired by the BioSpecimen Sharing Program, exhibit altered gene expression and increased oxidative stress,^83^ possibly causing retinal damage, degeneration, or remodeling.^84,85^ Other basic research is examining morphological, histological, and molecular changes in the brains and eyes of rats exposed to head-down tilt.^86^

Altogether, these examples are in the early stages of mechanistic understanding and countermeasure development. Already each has benefited from translational approaches. We suggest that a more coordinated programmatic effort of horizontal and vertical integration will accelerate countermeasure development. In the closing section, we provide suggestions of how enhanced translational research could materialize at NASA.

## Options for NASA’s future translational research path

Given the need for acute astronaut countermeasures to enable longer-duration missions, we recommend the following steps to catalyze translational research within NASA’s Life Sciences portfolio.

### Building and managing partnerships

Greater coordinated efforts in translational research are needed to understand and manage the integrated organismal response to spaceflight and enable human exploration. Current activities within NASA’s SLPSRA Division are creating new opportunities for translational science both within and external to the Agency. Tools like text analytics should aid in managing the life sciences portfolio and identify new gaps in knowledge. Other government agencies, such as NIH, and other organizations, both commercial and non-profit, have been working for a number of years on Earth-based translational research goals. NASA should partner, both nationally and internationally, with external organizations to benefit from their tools, techniques, and experience, and add NASA-unique data and translational research efforts to the much larger, continually growing, and worldwide community of translational research science.

### New collaborations

NASA’s Human Research and Space Biology programs should increasingly encourage translational research proposals for upcoming NASA research announcements (NRAs),^87^ including proposals that span traditional program boundaries or team science proposals (horizontally or vertically-integrated teams of investigators like the NSCORs or resembling the P01 Program Project Grants offered by NIH). Additionally, phased awards allowing progression from hypothesis-driven science to countermeasure development would enable vertical translation across NASA’s life sciences portfolio (Figs. 1, 2). Finally, proposals with international collaborations are encouraged to leverage the ISS platform and unique national resources (see the open NRAs and the International Space Life Sciences Working Group for research announcements).

### NASA’s open science and data sharing

Open Science approaches, allowing transparency and data sharing at all levels of society, are being developed at NASA to provide greater access to data and tissue sharing from both space and ground-based research. The goal is to engage researchers both within and outside of the NASA community (e.g., academia, NIH, Department of Defense, etc). These steps are essential for both optimizing the use of these valuable resources and facilitating translational research goals. NASA should continue the development and expansion of Open Science systems, such as GeneLab, to enable translational research for the broadest research community.

### Education, communications, and advocacy

Increased activity in the Education, Communication, and Advocacy areas will be fundamental to achieve translational research goals and is a mandate of the HRP Translational Research Institute. Additionally, organizations such as the ASGSR are discussing options to engage their members who span biology, human research, physical sciences, life support, related technology development, and commercial applications in translational research pursuits. NASA should develop research fellowships, as well as educational and scientific communication materials specifically tailored to the Agency’s translational research goals and partner with external organizations, wherever possible, to leverage the advocacy those organizations can provide.

## Closing

In summary, translation research is a two-phased effort at NASA and will help accelerate countermeasure development for exploration missions. We have specified four areas where synergy within the existing program structures seems promising and have suggested ways in which program coordination and team science approaches can facilitate translation from discovery to development to implementation.
